# Pulmonary wedge resection plus parietal pleurectomy (WRPP) versus parietal pleurectomy (PP) for the treatment of recurrent primary pneumothorax (WOPP trial): study protocol for a randomized controlled trial

**DOI:** 10.1186/s13063-015-1060-z

**Published:** 2015-11-30

**Authors:** Jens Neudecker, Uwe Malzahn, Peter Heuschmann, Uwe Behrens, Thorsten Walles

**Affiliations:** Department of General, Visceral, Vascular and Thoracic Surgery, Surgical Regional Centre Berlin of CHIR-Net, Charité University Medicine Berlin, Campus Charité Mitte, Charitéplatz 1, 10117 Berlin, Germany; Clinical Trial Center Würzburg (CTCW), University Hospital Würzburg, Josef-Schneider Strasse 2, 97080 Würzburg, Germany; Coordinating Center for Clinical Studies (KKS), Charité, Augustenburger Platz 1, 13353 Berlin, Germany; Department of Cardiothoracic Surgery, University Hospital Würzburg, Oberdürrbacher Strasse 6, 97080 Würzburg, Germany

**Keywords:** Recurrent primary pneumothorax, Parietal pleurectomy, Pulmonary wedge resection, Prospective randomized trial, Multicenter

## Abstract

**Background:**

For the surgical treatment of recurrent primary spontaneous pneumothoraces (rPSP) different operative therapies are applied to achieve permanent freedom from recurrence.

**Methods/design:**

This multicenter clinical trial evaluates the long-term results of two commonly applied surgical techniques for the treatment of rPSP. Based on the inclusion and exclusion criteria, and after obtaining the patients’ informed consent, participants are randomized into the two surgical treatment arms: pulmonary wedge resection plus parietal pleurectomy (WRPP) or parietal pleurectomy alone (PP). Consecutively, all study participants will be followed up for two years to evaluate the surgical long-term effect. The primary efficacy endpoint is the recurrence rate of pneumothorax within 24 months after surgery. The calculated sample size is 360 patients (*n* = 180 per treatment arm) to prove superiority of one of the two treatments. So far, 22 surgical sites have submitted their declaration of commitment, giving the estimated number of participating patients.

**Discussion:**

A prospective randomized clinical trial has been started to compare two established surgical therapies to evaluate the long-term results regarding recurrence rates. Furthermore, cost of treatment, and influence on the perioperative morbidity and mortality as well as on quality of life are analyzed. If the study reveals equivalence for both surgical techniques, unnecessary pulmonary resections could be avoided.

**Trial registration:**

ClinicalTrials gov: NCT01855464, 06.05 2013.

**Electronic supplementary material:**

The online version of this article (doi:10.1186/s13063-015-1060-z) contains supplementary material, which is available to authorized users.

## Background

### Rationale

A pneumothorax (PTX) is an accumulation of air in the pleural space between the lung and chest wall [1]:

- A **primary pneumothorax** (pPTX) occurs in healthy individuals without pre-existing lung disease and without previous thoracic intervention or chest injury.
- A **secondary pneumothorax** is usually the result of an underlying lung disease (for example, pulmonary emphysema) or a previous intervention (such as pleural puncture) or a thoracic trauma (both blunt and penetrating).

pPTX represents a common disease in industrialized countries with an annual incidence of 18–28 per 100,000 [2, 3]. The goal of surgical pneumothorax treatment is full re-expansion of the collapsed lung parenchyma and recurrence prevention. Varying surgical strategies are followed to achieve these targets. Thoracoscopic parietal pleurectomy is the most frequently used surgical technique. The benefit of additional resection of the pulmonary apex, however, is still questionable [4, 5]. A theoretical advantage might be a stronger adhesion between the stapled apex of the lung and the chest wall (for example, in the sense of a foreign body reaction), which could be accompanied by a lower recurrence rate. On the other hand, the use of stapling devices for pulmonary wedge resection increases surgical costs and the risk of postoperative bleeding and parenchymal fistulas, translating into longer hospital stays. However, both surgical strategies are routinely applied in Germany’s specialized thoracic surgery units. While their published short-term postoperative results seem to be comparable, long-term results are still missing. Furthermore, the associated perioperative morbidity and mortality and the postoperative quality of life have not been evaluated so far. In a recent observational study, it has been shown that up to 45 % of patients after operative therapy have recurrent and occasionally persistent postoperative symptoms such as scar pain and paresthesia [6]. Until now there has been no prospective randomized clinical trial on this topic. Retrospective cohort analyses describe both methods as equivalent [7]. The rationale of the study protocol for this prospective randomized multicenter study was to evaluate whether pulmonary wedge resection in combination with a partial apical pleurectomy (WRPP) is superior to the partial apical pleurectomy alone (PP) for treatment of recurrent pPTX.

## Methods/design

### Hypothesis

The WOPP (Wedge Resection or Parietal Pleurectomy for the Treatment of Recurrent Pneumothorax) study is a two-arm, prospective, randomized, nationwide multicenter clinical study in thoracic surgery, which was designed to test the hypothesis of whether partial pleurectomy for the treatment of recurrent primary pneumothorax alone is inferior compared to an additional wedge resection regarding the recurrence rate over two years.

### Inclusion and exclusion criteria

All patients between 15 and 40 years of age with recurrent pPTX and patients who have a therapy refractory first event of a pneumothorax are included. A treatment-resistant first event of a pneumothorax is considered to be a pPTX treated by insertion of a chest tube and PTX recurrence following chest tube removal during the same hospital stay. Excluded are patients with existing pulmonary fistula, patients with known underlying lung disease, and patients who have already undergone surgical or interventional pleurodesis or ipsilateral thoracic surgery. The latter does not include previously inserted chest tubes. The presence of single or multiple small or large bullae in the preoperative imaging or during thoracoscopy does not represent an exclusion criterion for patients.

### Ethical approval statement

The ethics committees of the University of Würzburg and all participating centers have approved the study protocol. A list of all ethical bodies that approved this study is provided in Additional file 1.

### Randomization

To prevent selection bias and insure against accidental bias, we will allocate patients to the treatment groups by randomization. In this way we can expect to get comparable study groups regarding observed and unobserved confounders.

**Fig. 1:**
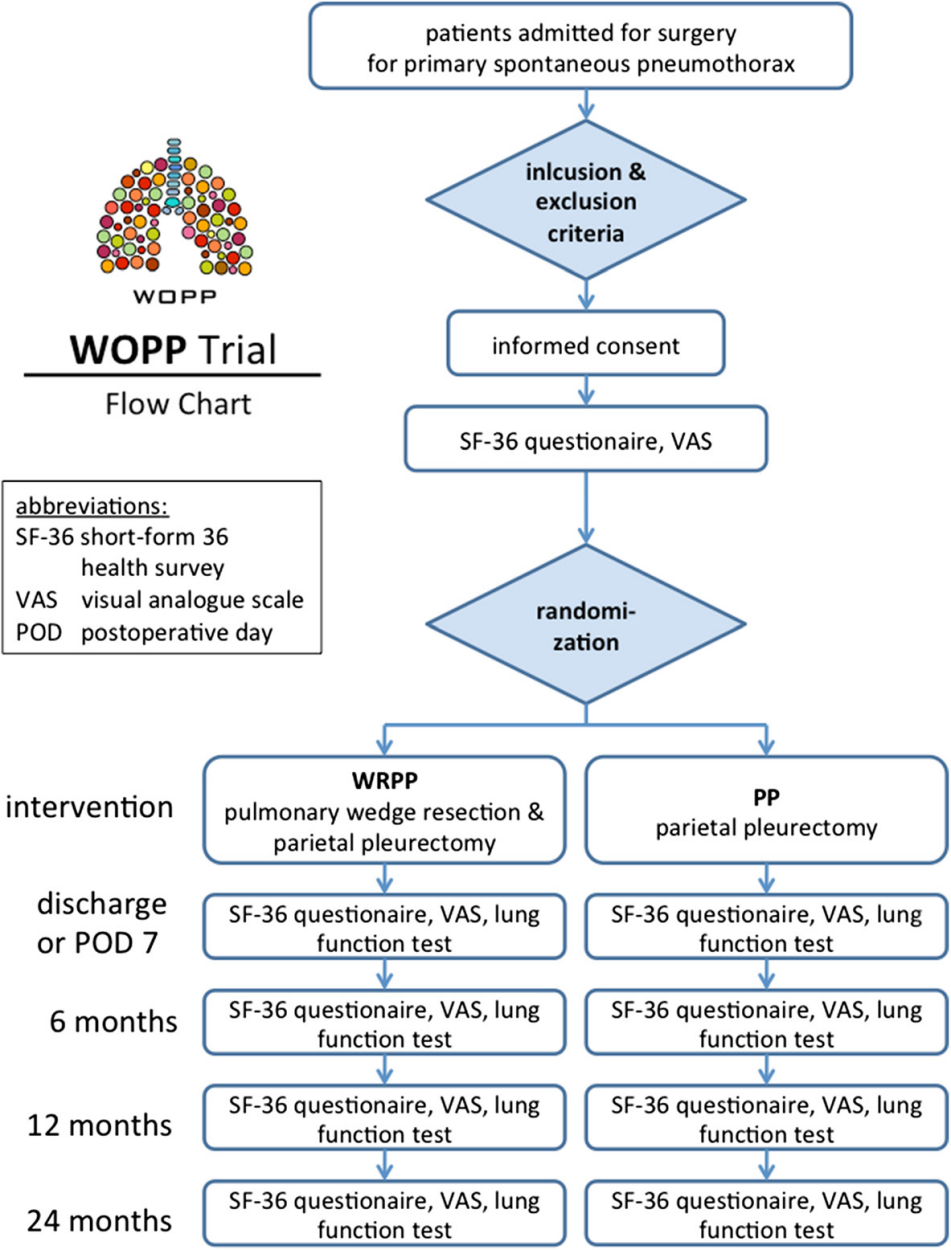
Course of the WOPP trial

Patients who are suitable after verification of the inclusion and exclusion criteria will be thoroughly informed about the WOPP study by a study physician. After approval of the study and submission of the written informed consent, patients are randomized. Randomization is done electronically via a web-based software tool (SecuTrial, interActive Systems GmbH, Berlin, Germany). We chose an allocation ratio of 1:1 because it maximizes statistical power for a given total sample size and there are no ethical or practical objections to this ratio. To minimize random error, 750 patients are to be screened and 360 patients (*n* = 180 per arm) will be randomized. Twenty centers have declared their participation in the study. The study design is shown in Fig. 1.

### Blinding

Blinding of the patient and the surgeon after randomization is not meaningful because staple lines after pulmonary wedge resection can often be seen on postoperative chest X-rays.

### Endpoints

The primary endpoint is PTX recurrence within the first 24 months after operative treatment. PTX recurrence is defined as postoperative lung collapse documented by chest X-ray. Secondary endpoints are morbidity, mortality, and postoperative convalescence. Morbidity will be differentiated between general and surgical complications. In addition, postoperative pain (at rest/under exertion) will be determined by the visual analogue scale (VAS). Postoperative quality of life will be captured by using the SF-36 questionnaire.

### Sample size estimation

The sample size calculation is based on the primary endpoint PTX recurrence within the first 24 months. We will test the null hypothesis of equal underlying population proportions for PTX recurrence for patients treated with pPTX alone and patients treated by WRPP by using a two-sided Fisher’s exact test at significance level 0.05.

Our assumptions for calculating the sample size are based on Czerny et al. [7]. Conservatively we assume underlying PTX recurrence proportions of 8.6 % for patients under pPTX and 1.4 % for patients under WRPP, a difference of 7.2 %. Sample sizes of 170 for both groups ensure a power of 80 % to detect this difference as a significant deviation from the null hypothesis of equal PTX recurrence proportions for both treatment strategies. Due to the visits without painful procedures and the comparatively young and healthy study population, we assume a low drop-out rate of 5 %. Therefore, 2*180 = 360 patients are to be included in the study at the beginning. PASS 11, developed by NCSS Statistical Software, Kaysville, UT, USA, was used for sample size calculation.

### Statistical analysis

The primary statistical analysis will be performed in the intention-to-treat (ITT) population. This analysis set includes all randomized subjects for which a diagnostic thoracoscopy has confirmed that thoracoscopic operation is feasible. We assume that the number of patients with recurrent pneumothorax after 24 months follows a binomial distribution in both groups. Within the primary analysis we will test the null hypothesis of equal proportions, which means equal probabilities for recurrent pneumothorax after 24 months for patients subject to WRPP and patients subject to PP by a two-sided Fisher’s exact test. Furthermore, a stratified analysis with the stratification factor study center will be performed using Mantel-Haenszel estimation of relative risk and testing for heterogeneity of stratum risks.

Within a secondary analysis we will calculate an adjusted odds ratio for the experimental treatment method WRPP relative to the comparing treatment PP using multiple logistic regression analysis. Secondary outcome analyses will be carried out depending on scale level and distribution and will be considered exploratory. In our analyses, 95 % confidence intervals will be calculated. The method for constructing the confidence interval in each case will depend on the type of the response variable and the variable to be tested. Controlling for potential confounders (such as age, gender, and smoking) will be performed by using multiple statistical models adjusting for these variables. Additional subgroup analyses considering gender aspects, age, and study center are planned. SAS 9.3 or corresponding subsequent SAS versions will be used for all data analyses.

### Study procedure

All patients with pPTX recurrence or a refractory first event will be screened for study participation. For each potential study participant the inclusion and exclusion criteria will be checked by a study physician. After providing informed consent, the participant will be randomized in one study group.

The baseline pain level and the quality of life are determined using the SF-36 questionnaire and the VAS score prior to any other study intervention in all participants. The standard treatment is parietal pleurectomy alone (control arm), and the validating therapy is the parietal pleurectomy in combination with an additional apical pulmonary wedge resection (experimental arm). All surgeries are performed by video-assisted thoracoscopy under general anesthesia and include a parietal pleurectomy for PTX treatment. Additionally, in the experimental group an apical pulmonary wedge resection is performed. The postoperative treatment is carried out according to the respective hospital standard. Perioperative variables like morbidity, mortality, subjective pain, duration of chest tube, surgical reintervention, and length of hospital stay will be determined. To assess the long-term effect of surgical intervention, all patients will be followed up for two years. The examinations for the individual follow-up visits are listed in Table 1.

**Table 1:** Overview of the study-related examinations

	Pre-OP	Intra-OP	Station	Follow-up		
Visit	V1		V2	V3	V4	V5
Week/month	OP-X		7. post OP day or day of discharge	6 months	12 months	24 months
Inclusion/exclusion criteria	X					
Consent	X					
Demographic data	X					
Medical history	X					
Smoking	X		X	X	X	X
Physical examination	X					
Chest X-ray^a^	X					
Pain level (VAS)	X		X	X	X	X
Quality of life (SF-36, German version)	X		X	X	X	X
Vanderschueren classification		X				
Postoperative complications			X	X		
AEs/SAEs			X	X	X	X
Duration of hospital stay			X	X		
Material costs^b^						X

^a^In case of pregnancy of a study participant, the chest X-ray will be replaced by a chest ultrasound

^b^At the end of the operation, the number of used trocars and stapling magazines will be documented

### Data management

The patient data are collected using electronic data entry screens (Case Report Forms, eCRFs). The study software complies with the regulatory requirements in accordance with GCP, FDA 21 CFR Part 11, and contains i) audit trail, ii) electronic signature, iii) programmable plausibility, consistency, and range checks, iv) query management system for online monitoring, and v) customizable roles and permissions system. In the study, software pseudonyms for the study data are used. This ensures the strict separation of identifying data (IDAT) and medical data (MDAT) of patients. Medical data are sent without personally identifiable information to the study database and saved there. However, any identifying patient data will be printed at the study center and remain there. The link between IDAT and MDAT can only be carried out by using a key, also called the pseudonym. Each study center maintains a confidential list of all patients, in which the patient numbers are connected with the full patient names. Only the local study team and the monitor will have access to this list. The original files can be viewed by monitors, auditors, and inspectors. The study database is protected by an authentication method. Only authorized study personnel with a personal identifier code will have access to the study database. Individual permission levels and roles are defined. This affords the implementation of different processing modalities of the study data, for example, to unlock certain forms (partial data) for editing, reading, or data control (review). The study database is permanently accessible via the Internet. The study data are recorded online and are transmitted directly into the study database; there is no local data storage. Data transfer between the local computer and the study database occurs via a secure connection (SSL encryption). To ensure data security, study data will be saved by daily backups of the study database. In addition, the availability of the study server is guaranteed by using the professional server environment at the IT center of the Charité University Medicine Berlin. To ensure the quality of the study data, the monitor performs data control during the study period. The originals of all central study documents including documentation forms are kept in the study coordination office (with the lead investigator) for at least ten years after completion of the study. The participating study centers keep the incurred administrative documents (correspondence with ethics committee, study management, study center), patient identification list, the signed consent forms, copies of the documentation forms, and the general study documentation (protocol, Amendments) for the above period. The original data of the study patients (medical records) are kept according to the filing deadline applicable to the study sites, but for no less than ten years.

### Monitoring

The coordination and implementation of the monitoring is performed by the Clinical Trial Center Würzburg (CTCW) at the University Hospital of Würzburg and the Surgical Regional Center (CRZ) Berlin. Here, the compliance with the requirements of the study protocol and ICH/GCP Guidelines are reviewed by the monitors. Each center is visited once before randomization of the first patient (initiation visit). Monitor visits are carried out at appropriate intervals during the study and at study completion. The verification of the correct transmission of data in the medical record into the documentation sheets (100 % Source Data Verification) is performed for the first two patients enrolled in each study center. The monitoring of the core data is performed for every patient. The following is considered as core data: existence of the patient, patient consent, correct inclusion and exclusion criteria, primary outcomes, and serious adverse events according to the protocol.

### Assessment of safety, analysis and reporting

Adverse events (AEs) have been defined as pneumonia, pleural effusion, postoperative bleeding requiring revision, blood loss requiring transfusion, pleural empyema, persistent lung fistula that last longer than five days after surgery, deep vein thrombosis, and wound infection. Serious adverse events (SAEs) are medical occurrences that lead to any of the following: immediately life-threatening situation, re-operation, persistent or significant disability/incapacity, inpatient hospitalization or prolongation of existing hospitalization, admission to an intensive care unit, death. All SAEs and all adverse events AEs are documented and assessed by the investigator and documented in the medical record and in the eCRF, regardless of whether, in the opinion of the investigator, there is a causal relationship with the study conduct or not. The documentation includes the type of event, start, end, expression/severity, causality, and outcome of the incident. Furthermore, the investigator must inform the lead investigator about the occurrence of an SAE within three days after having taking note of it and provide the investigator with a detailed written report (SAE sheet). Fatal or life-threatening SAEs will promptly, meaning within 24 hours, be reported after they are known.

### Data Safety Monitoring Board (DSMB)

An independent data protection committee (DSMB), which consists of three external experts (thoracic surgeon, biometrician, clinical scientist), will be concerned with patient safety and evaluation of benefits and risks. The DSMB examines the data collected and monitors patient safety by evaluation of AEs and SAEs. On the basis of the evaluated data, the DSMB makes recommendations on continuation, modification, or termination of the trial. The DSMB will meet regularly, at least five times during the duration of the study.

### Dropout/exclusions

The termination criteria of the study are defined as thoracic reintervention (for example, drainage system, thoracentesis with suction, surgery due to recurrence or other indication), the patient’s desire to withdraw from the current study, and violation of the protocol. During the hospital stay after surgery (and thus after randomization) a new or additional chest tube is not a termination criterion. Also, a pleural puncture for aspiration of pneumothorax or residual pneumothorax is not a termination criterion. Reoperation during the hospital stay after the initial procedure with the need for a parenchymal resection (for example, due to a recurrence pneumothorax or fistula formation) will cause the exclusion of the patient from the study. Patients may cancel their participation in the study at any time they wish, without giving reasons for the decision. In addition, the principal investigator may exclude a patient from the study if the continuation of the study compromises the patient’s well-being. The study cancellation will be documented in the eCRF and in the patient’s file, and all SAEs occurring from this point must be followed up. Premature discontinuation of the entire study will be done at the recommendation of the DSMB that the study be terminated for security reasons.

## Discussion

Here we report the design of a thoracic surgical multicenter study WOPP trial which compares two surgical procedures for the treatment of recurrent pPTX. Both surgical procedures, the partial apical pleurectomy alone (PP) and the partial apical pleurectomy in combination with an additional apical wedge resection (WRPP), are considered to be safe and well established in specialized thoracic surgery services worldwide [5, 7, 8]. The aim of this study is to evaluate whether parietal pleurectomy in combinination with apical wedge resection is superior compared to parietal pleurectomy alone in surgical treatment of pPTX. There is no prospective randomized study on this subject so far. Czerny et al. [7] reported a retrospective analysis of 113 patients, 45.2 % receiving partial pleurectomy (PP) and 54.8 % a partial pleurectomy with apical wedge resection (WRPP) with recurrence rates after PP of 7 % and after WRPP of 0 % (*p* = 0.009). The observation period was a mean of 38.7 months.

Although guidelines for the treatment of primary spontaneous pneumothorax exist, in daily clinical routine a variety of treatment methods are being used [9–14]. According to a recent AWMF guideline [15], indications for surgical intervention of pneumothorax are: first event with radiologically or thoracoscopically recognizable bullae, first recurrence after chest tube treatment, bilateral synchronous pneumothorax, tension pneumothorax, or hemopneumothorax. Video-assisted thoracoscopic surgery (VATS), if necessary thoracotomy, complete inspection of the lungs, closure of the leakage site or removal of the bubble-bearing areas of the lung, and a partial parietal pleurectomy or pleurodesis are recommended. While specialized thoracic centers rather focus on the guidelines, the majority of hospitals mostly perform partial pleurectomy alone [4, 5]. Finally, economic factors play an independent role, so that often the less expensive method is preferred [16]. Our multicenter study intends to evaluate the long-term results (recurrence rate over two years), perioperative morbidity and mortality, postoperative convalescence, and postoperative quality of life. Additionally, data will be collected that allow conclusions on the treatment costs of both surgical procedures. For data analysis a consultant health economist will be hired.

### Trial status

Adequate financial resources are available to cover appropriate personnel (study nurses, project coordinators, monitors, data managers) and additional expenses. The study protocol was approved by the Ethics Committee of the Medical Faculty of the University of Würzburg. The WOPP trial has been registered since 06.05.2013 on http://clinicaltrials.gov/ and can be identified worldwide by the assigned identification number NCT01855464.

All 20 participating study centers have received a study synopsis and signed the commitment letter (“Declaration of Commitment”). The currently (as of 07.01.2015) participating study centers are (see Table 2): Vivantes Clinic Neukölln (Berlin), DRK Clinic Berlin-Mitte, Protestant Lung Clinic Berlin, Charité Campus Mitte, University Hospital Erlangen, University Hospital Freiburg, Lung Clinic Großhansdorf, University Hospital Hamburg-Eppendorf, Thorax Clinic at the University Hospital Heidelberg, St. Bernward Hospital Hildesheim, Lung Clinic Cologne-Merheim, Clinic Lowenstein, University of Munich, Asklepios Clinic Munich-Gauting, Thorax Center district of Unterfranken, Hospital Barmherzige Brüder Regensburg, University Hospital Regensburg, Robert Bosch Hospital in Gerlingen, University Hospital of Tübingen, and the University Hospital of Würzburg.

**Table 2:** Participating trial centers

Hospital	Principal Investigator
Charité Universitätsmedizin Berlin, Klinik für Allgemein-, Visceral-, Gefäß- und Thoraxchirurgie	Priv.-Doz. Dr. med. Jens Neudecker
DRK Kliniken Berlin-Mitte, Klinik für Chirurgie und Thoraxchirurgie	Priv.- Doz. Dr. med. Paul Schneider
Evangelische Lungenklinik Berlin, Klinik für Thoraxchirurgie	Dr. med. Gunda Leschber
Vivantes Klinikum Neukölln, Thoraxchirurgie, Berlin	Dr. med. Stephan Eggeling
Universitätsklinikum Erlangen, Thoraxchirurgische Abteilung in der Chirurgischen Klinik	Prof. Dr. med. Horia Sirbu
Universitätsklinikum Freiburg, Abteilung Thoraxchirurgie	Prof. Dr. med. Bernward Passlick
Robert-Bosch-Krankenhaus, Klinik Schillerhöhe, Abteilung für Thoraxchirurgie, Gerlingen	Prof. Dr. med. Godehard Friedel
LungenClinic Großhansdorf, Thoraxchirurgie	Dr. med. Christian Kugler
Universitätsklinikum Hamburg- Eppendorf	Prof. Dr. med. Jakob Izbicki
St. Bernward Krankenhaus Hildesheim	Dr. med. Andreas Simon
Thoraxklinik am Universitätsklinikum Heidelberg	Prof. Dr. med. Hans Hoffmann
Kliniken der Stadt Köln gGmbH, Lungenklinik Köln-Merheim	Prof. Dr. med. Erich Stoelben
Klinik Löwenstein, Thorax- und Gefäßchirurgie	Priv.-Doz. Dr. med. Thomas Graeter
Asklepios Fachkliniken München-Gauting	Prof. Dr. med. Rudolf Hatz
LMU München, Klinik für Allgemeine, Viszeral-, Transplantations-, Gefäß- und Thoraxchirurgie	Prof. Dr. med. Rudolf Hatz
Thoraxzentrum Bezirk Unterfranken, Thoraxchirurgie, Münnerstadt	Dr. med. Boris Kardziev
KH Barmherzige Brüder Regensburg	Prof. Dr. med. Hans-Stefan Hofmann
Universitätsklinikum Regensburg, Herz-, Thorax- und herznahe Gefäßchirurgie	Prof. Dr. med. Hans-Stefan Hofmann
Universitätsklinikum Tübingen, Klinik für Thorax-, Herz- und Gefäßchirurgie	Dr. med. Volker Steger
Universitätsklinikum Würzburg, Klinik und Poliklinik für Thorax-, Herz- und Thorakale Gefäßchirurgie	Prof. Dr. med. Walles

Based on the declaration of commitment of all centers, more than 500 patients should be included in a period of 24 months in the study. Each study center plans to recruit annually between 8 and 20 patients. University and non-university hospitals will take part in the study to increase the generalizability of the study results. All participating centers are familiar with the conduct of clinical trials. Four of the study sites belong to the national CHIR-*Net* network of surgical regional study centers, which are experienced in prospective randomized multicenter trials. The final study protocol was submitted to all the centers. The first version of the electronic Case Report Form (eCRF) and the randomization tool exist. Recruitment was started in October 2013. Currently (as of 22.01.2015), there are 61 patients randomized. The end of recruitment is planned for October 2015 (“last-patient-in”), and the last study visit will be carried out until November 2017.

### Good clinical practice

The procedures that are defined in the study protocol, which relate to the implementation, evaluation, and documentation of the study, are designed to ensure that all persons who are involved in the study correspond to good clinical practice and the ethical principles described in the Declaration of Helsinki. The study will be conducted in accordance with local legal and regulatory requirements [17–19].

## Trial status

Electronic Case Report Forms have been created. Patient recruitment started in September 2013 with the centrally organized initiation meeting on 17.09.2013 in Berlin. Enrollment of the First Patient In was on 19.11.2013.

## Additional file

Additional file 1:
**List of participating centers and ethical bodies.** (DOCX 5 kb)

## Abbreviations

ITT: intention to treat
PP: parietal pleurectomy
pPTX: primary pneumothorax
PTX: pneumothorax
rPSP: recurrent primary spontaneous pneumothorax
VAS: visual analogue scale
WRPP: wedge resection plus parietal pleurectomy

## References

[1] Henry M, Arnold T, Harvey J (2003). BTS guidelines for the management of spontaneous pneumothorax. Thorax.

[2] Melton LJ, Hepper NCG, Offord KP (1979). Incidence of spontaneous pneumothorax in Olmsted County, Minnesota: 1950–1974. Am Rev Respir Dis.

[3] Bense L, Wiman LG, Hedenstierna G (1987). Onset of symptoms in spontaneous pneumothorax: correlations to physical activity. Eur J Respir Dis.

[4] Chan JW, Ko FW, Ng CK, Yeung AW, Yee WK, So LK (2009). Management of patients admitted with pneumothorax: a multi-centre study of the practice and outcomes in Hong Kong. Hong Kong Med J.

[5] Gossot D, Galetta D, Stern JB, Debrosse D, Caliandro R, Girard P (2004). Results of thoracoscopic pleural abrasion for primary spontaneous pneumothorax. Surg Endosc.

[6] Sostheim UA. Operative management of spontaneous pneumothorax: long term results at the Schillerhöhe hospital. URL: https://publikationen.uni-tuebingen.de/xmlui/handle/10900/46063 (German)

[7] Czerny M, Salat A, Fleck T, Hofmann W, Zimpfer D, Eckersberger F (2004). Lung wedge resection improves outcome in stage I primary spontaneous pneumothorax. Ann Thorac Surg.

[8] Ackermann C (1985). 10-year experience in parietal pleurectomy for treatment of pneumothorax. Helv Chir Acta.

[9] Chambers A, Scarci M (2009). In patients with first-episode primary spontaneous pneumothorax is video-assisted thoracoscopic surgery superior to tube thoracostomy alone in terms of time to resolution of pneumothorax and incidence of recurrence?. Interact Cardiovasc Thorac Surg.

[10] Kelly AM, Kerr D, Clooney M (2008). Outcomes of emergency department patients treated for primary spontaneous pneumothorax. Chest.

[11] Hürtgen M, Buhr J, Schwemmle K, Linder A, Friedel G (1996). Video-assisted thoracoscopic surgery for spontaneous pneumothorax — results after 4 years. Min Invas Ther Allied Technol.

[12] Hürtgen M, Linder A, Friedel G, Toomes H (1996). Videoassisted thoracoscopic pleurodesis. A survey conducted by the German Society for Thoracic Surgery. Thorac Cardiovasc Surg.

[13] Hürtgen M, Linder A, Friedel G, Toomes H. [Determination of established techniques for pleurodesis for pneumothorax treatment]. Zentralbl Chir 1997; 122(8);628–32 (German). 9412091

[14] Steger V, Walles T, Walker T, Friedel G (2007). Long-term follow-up of thoracoscopic talc pleurodesis for primary spontaneous pneumothorax. Eur Respir J.

[15] [Primary and secondary pneumothorax] In: AWMF online. http://www.awmf.org/leitlinien/detail/anmeldung/1/ll/010-007.html (German). Accessed 14 October 2015.

[16] Anheier H (2010). Economic constraints in the hospital: Do not accept without resistance. Deutsch Ärztebl.

[17] WMA Declaration of Helsinki - Ethical Principles for Medical Research Involving Human Subjects. In: World Medical Association. http://www.wma.net/en/30publications/10policies/b3/. Accessed 21 October 2015.

[18] Eudralex Volume 10 Guidelines on Good Clinical Practice (ICH E6: Good Clinical Practice: Consolidated guideline, CPMP/ICH/135/95. In: European Compliance Academy (ECA). http://www.gmp-compliance.org/eca_guideline_3361.html. Accessed 21 October 2015.

[19] Directive 2001/20/EC of the European Parliament and of the Council of 4 April 2001 on the approximation of the laws, regulations and administrative provisions of the Member States relating to the implementation of good clinical practice in the conduct of clinical trials on medicinal products for human use. http://eur-lex.europa.eu/legal-content/EN/TXT/HTML/?uri=CELEX:32001L0020&from=DE. Accessed 21 October 2015. 16276663

