# Supplementary material for: Pulmonary wedge resection plus parietal pleurectomy (WRPP) versus parietal pleurectomy (PP) for the treatment of recurrent primary pneumothorax (WOPP trial): study protocol for a randomized controlled trial
Source: Trials. 2015 Nov 30;16:540. doi: 10.1186/s13063-015-1060-z (PMC4663732; doi:10.1186/s13063-015-1060-z)
Supplement: Additional file 1: — List of participating centers and ethical bodies. (DOCX 5 kb) [file 13063_2015_1060_MOESM1_ESM.docx]

Additional file 1

List of participating centres and ethical bodies

| **Participating Institution** | **Responsible Ethical Committee** | **Reference Number** |
| --- | --- | --- |
| Würzburg University Hospital  Oberdürrbacher Str. 6 97080 Würzburg | =First Vote  Ethics Committee of the Faculty of Medicine,  University Würzburg  Institute for Pharmacology and Toxicology  Versbacher Str. 9  97078 Würzburg | 29/13 |
| Charité - Universitätsmedizin Berlin Chariteplatz 1 10117 Berlin | No Second Vote  *First Vote from leading Ethics Committees has been accepted* | 29/13 |
| Red Cross Hospital Berlin Drontheimer Straße 39–40 13359 Berlin | No Second Vote  *First Vote from leading Ethics Committees has been accepted* | 29/13 |

| Prostestant Lung Hospital Berlin  Evangelische Lungenklinik Berlin Lindenberger Weg 27 13125 Berlin | No Second Vote  *First Vote from leading Ethics Committees has been accepted* | 29/13 |
| --- | --- | --- |
| Vivantes-Clinic Neukölln  Rudower Str. 48 12351 Berlin | No Second Vote  *First Vote from leading Ethics Committees has been accepted* | 29/13 |
| University Hospital Erlangen Krankenhausstr. 12 91054 Erlangen | Ethics Committee of the Faculty of Medicine,  University Friedrich-Alexander,  Erlangen-Nürnberg  Krankenhausstraße 12  91054 Erlangen | 234_13 Bc |
| University Hospital Freiburg Hugstetter Straße 55 79106 Freiburg | Ethics Committee of the University,  Albert-Ludwigs- University, Freiburg  Engelberger Straße 21  79106 Freiburg | 408/13 |
| Robert-Bosch Hospital  Schillerhöhe  Solitudestr. 18 70839 Gerlingen | Ethics Committee of the Faculty of Medicine,  Eberhard-Karls- University and Universtiy Hospital Tübingen  Gartenstraße 47  72074 Tübingen | 491/2013BO1 |
| LungenClinic Großhansdorf Wöhrendamm 80 22927 Großhansdorf | Ethics Committee of the regional Medical Association  Schleswig-Holstein  Bismarckallee 8-12  23795 Bad Segeberg | IV/EK |
| Thoraxklinik at Heidelberg University Hospital Amalienstr. 5 69126 Heidelberg | Ethics Committee of the Faculty of Medicine Heidelberg  Alte Glockengießerei 11/1  69115 Heidelberg | S-514/2013 |
| Lung Clinic  Krankenhaus Köln-Merheim - Ostmerheimer Str. 200 51109 Köln | Ethics Committee of University  Witten/Herdecke  Alfred-Herrhausen-Str. 50  58448 Witten | 109/2013 |
| Hospital of the  Ludwig-Maximilians-University (LMU) Munich Marchioninistraße 15 81377 München | Ethics Committee- LMU München  Pettenkoferstr. 8a  80336 München | 507-13 |
| Thoraxzentrum Bezirk Unterfranken Michelsberg 1 97702 Münnerstadt | No Second Vote  *First Vote from leading Ethics Committees has been accepted* | 29/13 |
| Barmherzige Brüder Hospital  Prüfeninger Straße 86 93049 Regensburg | Ethics Committee of University  Regenburg  93042 Regensburg | 13-103-0213 |
| University Hospital Regensburg Franz-Joseph-Strauß-Allee 11 93053 Regensburg | Ethics Committee of University  Regenburg  93042 Regensburg | 13-103-0213 |
| University Hospital Tübingen Hoppe-Seyler-Straße 3  72076 Tübingen | Ethics Committee of the Faculty of Medicine,  Eberhard-Karls- University and for  Universtiy Hospital Tübingen  Gartenstraße 47  72074 Tübingen | 491/2013BO1 |
| Asklepios Fachkliniken München-Gauting Robert-Koch-Allee 2 82131 München-Gauting | Ethics Committee- LMU München  Pettenkoferstr. 8a  80336 München | 507/13 |
| St. Bernward Hospital Treibestr. 9 31134 Hildesheim | Ethics Committee of the regional Medical Association  Niedersachsen  Berliner Allee 20  30175 Hannover | Grae/087/2014 |
| University Hospital Hamburg-Eppendorf Klinik für Allgemein-, Visceral-, Gefäß- und Thoraxchirurgie Martinistraße 53 20246 Hamburg | Ethics Committee of the regional Medical Association  Hamburg  Weidestraße 122 b  22083 Hamburg | MC-082/14 |
| Asklepios Clinic Langen  Viszeral- und Thoraxchirurgie  Röntgenstrasse 20  63225 Langen | Ethics Committee of the Regional Medical Association  Hessen  Im Vogelsgesang 3  60488 Frankfurt | MC 166/2015 |
| St. Elisabethen Hospital  Katharina Kasper-Kliniken  Thoraxchirurgie  Ginnheimer Str. 3  60487 Frankfurt am Main | Ethics Committee of the regional Medical Association  Hessen  Im Vogelsgesang 3  60488 Frankfurt | MC 166/2015 |
